# Disrupting the Status Quo: The New Normal of Family Caregivers’ Service Use

**DOI:** 10.1093/geroni/igab046.1367

**Published:** 2021-12-17

**Authors:** Karen Moss, Verena Cimarolli, Karen Rose

## Abstract

Family caregivers are essential partners in chronic disease management for older adults. However, being a family caregiver can have negative mental and physical health consequences, making it important for some caregivers to rely on supportive services, either for themselves (e.g. support groups) or to get help with caregiving tasks (e.g. home care). Supportive service use by family caregivers is well documented; yet, this research has often not included specific subgroups of caregivers (e.g. the racially/ethnically, or geographically diverse). Hence, the purpose of this symposium is to share new findings from research on supportive service use in understudied caregiving populations. First, Dr. Cimarolli presents findings from a study on the types of supportive services long-distance caregivers use for themselves and the factors associated with supportive service use in this caregiver population. Then, Dr. Wyman reports findings from a survey on the use of home and community-based resources by family caregivers in a Native American community. Dr. Wright will share the results of a systematic review of self-care interventions designed for caregivers of African Americans living with dementia. Finally, Dr. Mavandadi presents the results of a study examining the effectiveness of a telephone-based, collaborative dementia care program for improving outcomes in caregivers of military veterans living with dementia. Dr. Karen Rose will discuss the implications of each of these study findings for the development and evaluation of supportive interventions for these specialized family caregiver groups.

